# OP83 Uncovering The Hidden Evidence: Harnessing Perplexity Artificial Intelligence To Revolutionize Grey Literature Retrieval In Health Technology Assessment

**DOI:** 10.1017/S0266462325101372

**Published:** 2025-12-29

**Authors:** Lisa Tjosvold, Dagmara Reimer, Janice Kung

## Abstract

**Introduction:**

Health technology assessments (HTAs) demand exhaustive evidence retrieval, encompassing both peer-reviewed research and often elusive and poorly indexed grey literature, to guide informed healthcare decisions. This case study investigates Perplexity, a generative artificial intelligence (AI)-powered search engine, as a novel tool for uncovering diverse, often overlooked grey literature sources crucial for comprehensive HTA.

**Methods:**

Iterative searches in Perplexity were conducted utilizing a diverse range of tailored prompts to identify evidence sources, including organizational reports, guidelines, epidemiological data, statistical analyses, and white papers. Search results were evaluated for relevance and uniqueness, with comparisons made against traditional retrieval methods. Feedback from participating information professionals provided additional insights into Perplexity’s strengths, limitations, and overall usability. Furthermore, the characteristics of effective prompt engineering strategies were explored and methods for integrating Perplexity into evidence retrieval workflows for HTA were examined.

**Results:**

The results obtained through the Perplexity approach revealed several unique sources that were not identified using traditional search methods. Furthermore, the time required to locate and retrieve these distinctive sources was significantly reduced, making the process more efficient while broadening the scope of information gathered.

**Conclusions:**

This study showcased Perplexity’s potential to transform gray literature searches for HTAs. By efficiently uncovering diverse, hard-to-find sources, including those often missed by traditional methods, Perplexity enhanced both relevance and uniqueness, while also saving significant time in the search process. Insights from this study will underscore its value in optimizing evidence retrieval workflows, making HTAs more comprehensive, efficient, and impactful.

